# Convolution model for COVID-19 rate predictions and health effort levels computation for Saudi Arabia, France, and Canada

**DOI:** 10.1038/s41598-021-00687-8

**Published:** 2021-11-22

**Authors:** Yas Al-Hadeethi, Intesar F El Ramley, M. I. Sayyed

**Affiliations:** 1 Physics Department, Faculty of Science, King Abdulaziz University, Jeddah, 21589 Saudi Arabia; 2 INToo Solutions, 202-6940 East Blvd, Vancouver, BC V6M 3V5 Canada; 3 Department of Physics, Faculty of Science, Isra University, Amman, Jordan; 4 Department of Nuclear Medicine Research, Institute for Research and Medical Consultations (IRMC), Imam Abdulrahman Bin Faisal University (IAU), P.O. Box 1982, Dammam, 31441 Saudi Arabia

**Keywords:** Biological techniques, Diseases, Mathematics and computing

## Abstract

Many published infection prediction models, such as the extended SEIR (E-SEIR) model, are used as a study and report tool to aid health authorities to manage the epidemic plans successfully. These models face many challenges, mainly the reliability of the infection rate predictions related to the initial boundary conditions, formulation complexity, lengthy computations, and the limited result scope. We attribute these challenges to the absence of a solution framework that encapsulates the interacted activities that manage: the infection growth process, the infection spread process and the health effort process. In response to these challenges, we formulated such a framework first as the basis of our new convolution prediction model (CPM). CPM links through convolution integration, three temporal profile levels: input (infected and active cases), transformational (health efforts), and output functions (recovered, quarantine, and death cases). COVID-19 data defines the input and output temporal profiles; hence it is possible to deduce the cumulative efforts temporal response (CETR) function for the health effort level. The new CETR function determines the health effort level over a period. Also, CETR plays a role in predicting the evolution of the underlying infection and active cases profiles without a system of differential equations. This work covers three countries: Saudi Arabia, France, and Canada.

## Introduction

The world community went through an epidemic crisis like COVID-19 in 2020, like the "Spanish Flu" in 1918–1919 and SARS-1 in 2003. Despite considerable health advances, most countries seem to have been overwhelmed by the epidemic growth mainly due to the lack of a vaccine besides lagging health management that proved infective to ensure the quick end of the crisis. Health management is about the mechanics of collecting COVID-19 data and getting enough resources to absorb the sudden surges in infection rate and know ahead of time if the health plans and their implementation are enough to bring the infection to a quick end. Good health plans need daily data reports and qualitative and predictive modelling tools to enable health management to correctly navigate the infection diminishing path. The epidemic cycle modelling tools are statistical or mechanistic^1^. Such tools take the available epidemic information as an input and, after applying the initial boundary conditions and constraints, if any, to produce infection growth predictive reports as an output. In this paper, we will refer to the initial boundary conditions as initial conditions^2^ presented a list of models based on the solution schema to analyse the dynamics of COVID-19 and predict the effects of health control efforts on the spread of this virus in many countries worldwide. These models include the generalised logistic growth model; the discrete generalised logistic model; the exponential growth model; the Richards growth model; the discrete generalised Richard model; the generalised growth model; the discrete generalised growth model; the neural network-based quarantine control model, the sub-epidemic wave model, the SIR model, and the SEIR model.

Chinazzi Matteo et al. in^3^ utilised a global metapopulation model of disease transmissibility to devise the effect of travel restrictions on inland and global outbreak. At the onset of Wuhan's travel prohibition on January 23rd, 2020, the model showed that many infected travellers had already spread out in multiple cities in China. Modelling results indicate that although travel restrictions sustained 90% to and from mainland China. These restrictions had only moderately affected the epidemic course, and these restrictions must be combined with at least a 50% reduction of transmission in the community^3^. Furthermore^4–8^ utilised the global epidemic and mobility model (GLEAM), an individual-based, stochastic, and spatial epidemic model, for modelling the international prevalence of the COVID-19 outbreak. This GLEAM model deals with a metapopulation network integrated with real-world data at which the globe is split into subpopulations centred around major transportation hubs.

A statistical model introduced by Yifan Zhu and Ying Qing Chen^8^ concerning COVID-19 transmissibility relied on symptom appearance data for evaluating the disease transmission at the early-stage flare-up among the Chinese population. This model also provided sensitivity analyses with a variety of hypotheses regarding the natural history of the disease. Moreover, a model for symptom prediction of COVID-19 was presented by Menni et al.^9^, which gave a linear model of the disease-related symptoms such as cough, males, etc. Zhang et al.^10^ introduced a time-dependent SEIR model for fitting and predicting the time series of COVID-19 evolution monitored for three months in some provinces of China. The implementation of such a model demonstrated the viral transmission/infection rate based on space and the significance of space–time variation in the recovery rate. The validated SEIR model was then utilised to predict COVID-19 evolution in Japan, South Korea, the USA and Italy, countries that responded diversely to monitoring and moderating COVID-19. However, the previous predictions are highly uncertain because of the intrinsic alteration of the maximally infected people and the infection/recovery rates within various states. Moreover, a stochastic model based on the random walk particle tracking scheme, comparable to a mixing-limited bimolecular reaction model, was developed for evaluating non-pharmaceutical COVID-19 prevalence mitigating strategies. Applying the stochastic model, initiating tests demonstrated that self-quarantine might not be as effective as strict social distancing in decelerating COVID-19 occurrence.

Another group: the COVID, I. H. M. E Forecasting Team^11^ reported, using the SEIR model with data of COVID-19 case morbidity and mortality from February 1st, 2020 till September 21st, 2020 for modelling the probable causes of disease and the efficacy of non-pharmaceutical management in the USA. The assessment of mask use and social distancing policy was undertaken. The findings indicate that universal mask-wearing (by at least 95% of the public) can sufficiently effectively mitigate the impact of disease resurgences in various states. Kennedy et al.^12^ extended the SEIR compartmental model to represent the impact of limiting COVID-19 transmission strategies, the personal protection measures, continuous, intermittent, and stepping-down social distancing policy. They reported that this method effectively prevents a second peak and keeps the daily requirement for intensive care units within the limit of units currently available.

Interestingly, Neto et al.^13^ collected data from WHO presented a generalised (SEIR) compartmental model and examined it utilising a global optimisation algorithm. The SEIR model could precisely fit the data of either the active cases or deaths of all countries tested, applying optimised coefficient values in agreement with recent reports.

The model prediction reports, directly and indirectly, influence health policymakers at federal, state/province and regional levels. In our view, we can classify the following predictive models in current use as:

1. Epidemic Models or Mechanistic Models^14,15,28,29,31^ explore the epidemic cycle's fundamental bases and long-term trends, consider possible non-linear effects, and examine the role of equation control parameters. An example is the SEIR and the extended SEIR (E-SEIR)^16–25^. The studies in^28,29,31^ explicitly showed the influence of the authorities' efforts at various state levels on controlling COVID-19 spread at regional, provincial and state levels.
2. Agent-Based Models (ABM) approach is designed to model the epidemic spread process using Agent-Based Simulations (ABS) to simulate the pandemic dynamics collectively utilising a set of agents emulating individuals, enterprises, and government. Silva et al.^26^ developed an ABM to simulate the dynamics of the COVID-19 pandemic and the economic and epidemiological influences of social distancing measures. The objective was to simulate a closed society inhabiting an everyday ambience composed of population, residences, governmental units, healthcare systems, and business venues; each represents specific characteristics, stochastic behavioural attitudes, and many variable interactions. ABS's selection to simulate such systems is due to their ease of application and high accuracy compared to actual data^27^. ABS's primary objective is to simulate the storing statistics derived from the agents' internal states in every iteration, the system's temporal evolution, and the universal manners developing from the iterative agent interactions.
3. Statistical Models, which extrapolate the curves previously fitted on actual data, aim at providing short-term forecasts limited by data uncertainties whose impacts could be amplified by the highly non-linear nature of the governing relationships. In^30^, the team conducted a quantitative analysis to explore the role of travel restrictions and health control measures throughout the first 50 days of the COVID-19 epidemic in China. The study showed that the imposed health control efforts were associated with reductions in case prevalence in such a way that these efforts appear to have delayed the growth and limited the size of the COVID-19 epidemic in China.

We used the published COVID-19 epidemic statistics with an October 15th, 2020 cut-off date for three countries: Saudi Arabia, France, and Canada^14^. The actual data is a COVID-19 Data Repository collected by the Center for Systems Science and Engineering (CSSE) at Johns Hopkins University. This data includes the infected population rate *I(t)*, active case population rate *A(t)*, recovered population rate *R(t)*, and death population rate *D(t)*. The main objective of our work is to introduce the convolutional method to compute:

1. the cumulative health efforts temporal profile *f(t)* through the deconvolution of
  - the Fourier Transform (FT) of the infection population temporal profile, *I(t)* and the FT of the recovered population temporal profile *R(t)*, and
  - the FT of the active cases population temporal profile *A(t)* and the FT of the recovered population temporal profile *R(t)*,
2. the prediction for *I(t)* and *A(t)* utilising the time-extended version of *f(t),* which forms the basis of the CPM model. To establish the validity of this new approach, we presented a comparison between the CPM model and the E-SEIR. The model comparison is in terms of:
  - required resources,
  - efforts/steps,
  - complexity, and
  - results (artefacts and peak prediction).

“SEIR and E-SEIR models—an overview” Section  provides a brief overview of the SEIR and E-SEIR models. In this paper, we used the E-SEIR model as a starting point to set a benchmark with which we compared the result from our new modelling approach. “E-SEIR model computational results and discussion” utilises Python Differential Evolution (DE) as a solver for the E-SEIR model equations applicable to Saudi Arabia, France, and Canada. Then executed the necessary steps to produce an extended prediction of 60 days rather than just 30 days used by^9^. Also, in “E-SEIR model computational results and discussion”, we explored the role of the controlling/weighting parameters that are influenced by the set of initial conditions. “Epidemic process framework components” presents the epidemic process framework in which we identified three processes: Infection Spread Process (ISP), Infection Growth process (IGP), and Health Effort Process (HEP). Based on this, “Convolutional prediction model (CPM)—new proposal” shows the implementation of the SEIR transition stages in a new format to conclude the necessary set of cumulative efforts temporal response (CETR) functions. We used these functions to define the collective HEP at federal, state/provincial, regional, and individual levels that influence the SEIR stage populations. Then we used these CETR functions to formulate the CPM to predict the future numbers of the infected and active case populations. Each CETR function represents all-controlling measures such as lockdown policies, public health controls, and protective/hygiene individual behaviour, such as wearing face masks. Hence, our new model is not just to predict numbers but also helps to manage the COVID-19 crisis. Section “Discussion and conclusion” presents our conclusion regarding E-SEIR and CPM models and the potential future of updates.

## SEIR and E-SEIR models—an overview

**Figure 1:**
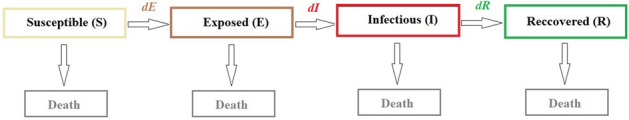
SEIR model transitional stages^21^.

The Susceptible-Exposed-Infection-Recover (SEIR) is a staged loop model in epidemiology, which assigns individuals into different transitional stages of illness (Fig. 1.)

The SEIR model transitions patients between four stages:

- Susceptible (**S**): the individuals who are exposed to the virus but not infected yet.
- Exposed (**E**): the individuals who are infected but not yet contagious. They could be in self-isolation or quarantine.
- Infectious (**I**): the individuals who are infected and contagious^2^.
- Recovered (**R**): the individuals who have recovered and are immune^2^.

Besides the above SEIR stage definitions, there are also assumptions, the most important of which is that population (N) is constant where the death rate equals the birth rate. All newborns are susceptible by default^23^. The transition dynamics between SEIR stages are governed by a system of first-order differential equations (SoDE)^9^ plus specific initial conditions:

1 $$\frac{dS(t)}{dt}= -\beta I\left(t\right)\frac{S\left(t\right)}{N}$$

2 $$\frac{dE(t)}{dt}= \beta I\left(t\right)\frac{S\left(t\right)}{N}- \gamma E(t)$$

3 $$\frac{dI(t)}{dt}= \gamma E\left(t\right)-(\lambda +\kappa )I(t)$$

4 $$\frac{dR(t)}{dt}= (\lambda +\kappa )I(t)$$

where the parameters β, γ, λ, and κ have (1/day) as a unit of measurement. β is called the infection rate. γ is the inverse of the average latent time and governs the lag between having undergone an infectious contact and showing symptoms: in the equations, it brings people from the E stage to the I stage. λ and κ are the recovery rate and the death rate, respectively. They give information about how fast the people may recover from the disease (1/λ is the average recovery time) and how many of them, unfortunately, die^20^. These four differential equations might not be accurate enough to describe COVID-19 stage transitions. In the SEIR Model, the asymptomatic patient will be considered as part of the infection count. A recovered patient ***R****(t)* may not be immune to COVID-19. Of course, with more robust evidence, it will be possible to reveal the traits of COVID-19^21^. From Fig. 1 and Eq. (4), the parameters for R (i.e. λ) and D (i.e. κ) are coupled together as output from the I stage. This assumption is one of the SEIR model framework formulation foundations^9^. Our interpretation is that λ and κ are the weighing parameters to bring people from the I stage to the R and D stages as linearly additive parameters rather than cumulative ones. Hence, stage R is the one bucket for recovered and dead populations, not just recovered. This assumption made the SEIR model insufficient for the COVID-19 evaluation cycle. Hence, some modification needs to be introduced to the model to describe the virus spread accurately. The SIR model may be more appropriate if the disease is more like measles, where a patient immediately becomes infectious. But, if the condition is more like flu, a recovered patient may still get re-infected later, then the **SEIR** model^21^ may be more relevant.

Given the limited knowledge of COVID-19, different variations of the SoDE for SEIR model equations have been implemented. The variation covers the equations, parameters, or different computational fitting techniques to make the model represent reality as accurately as possible. A generalised SEIR model^20^ has been adopted, which is based on^21^ to simulate the Italian situation, by relying on the following SoDE:

6 $$\frac{dS(t)}{dt}= -\beta \left(t\right)I\left(t\right)\frac{S\left(t\right)}{N}- \alpha \left(t\right)S(t)$$

7 $$\frac{dP(t)}{dt}= \alpha \left(t\right)S(t)$$

8 $$\frac{dE(t)}{dt}= -\beta \left(t\right)I\left(t\right)\frac{S\left(t\right)}{N}- \gamma E(t)$$

9 $$\frac{dI\left(t\right)}{dt}= \gamma E\left(t\right)- \delta I\left(t\right)$$

10 $$\frac{dQ(t)}{dt}= \delta I\left(t\right)-\lambda \left(t\right)Q\left(t\right)- \kappa \left(t\right)Q\left(t\right)$$

11 $$\frac{dR(t)}{dt}= \lambda \left(t\right)Q\left(t\right)$$

12 $$\frac{dD(t)}{dt}= \kappa \left(t\right)Q\left(t\right)$$

The above set of differential equations (Eqs. 6–12) is the expanded version used by^21^, which we will refer to in this paper as the E-SEIR model. The parameters in the above equations are as follows: alpha (α) is the protection rate, beta (β) is the infection rate, gamma (ϒ) is the inverse of the average latent time, delta (δ) is the rate at which infectious people enter quarantine, lambda (λ) is the time-dependent recovery rate, and kappa (κ) is the time-dependent mortality rate.

To establish a comparison benchmark with our new CPM model, we needed to solve Eqs. (6-12) above. We uploaded the available COVID-19 data for Saudi Arabia, France, and Canada. We used Python libraries for Differential Evolution (DE) method to numerically solve the equations by determining the parameters that optimise the solution and guarantee safe and best fit.

## E-SEIR model computational results and discussion

This section will demonstrate the necessary steps to build an E-SEIR model using a numerical solution approach for Eqs. (6-12) and the risks that might lead to an unreliable model. First, we need to assume distinct sets for the initial conditions associated with each of the E-SEIR stages' population. In this study, we are using two sets. Second, we need to define the relevant value limits on each parameter in Eqs. (6-12) to satisfy the conservation of the overall population N. In this work, we used the value range 0 to 1 for each parameter. Such constraints could influence the optimum solution surface. Third, we select the right mathematical package to execute the numerical computation which will produce the optimum or near optimum model solution and extract the solution parameters values for parameters α, β, γ, δ, λ, and κ. It is worth mentioning that most of these packages internally include curve smoothing, which typically uses the entire data grid length as a smoothing window, consequently leading to a one-peak model profile that lacks specific incremental behaviours. Fourth, we deduce the model predictions for each initial boundary condition set for each country. The three countries' model computation produces eighteen plots (each is a family of curves), six tables including two tables of observation summary each for a specific initial boundary condition. Using the data for the three countries Saudi Arabia, France, and Canada, allows us to explore the E-SEIR model trends within different environments.

### E-SEIR model computation for two sets of initial conditions

**Figure 2:**
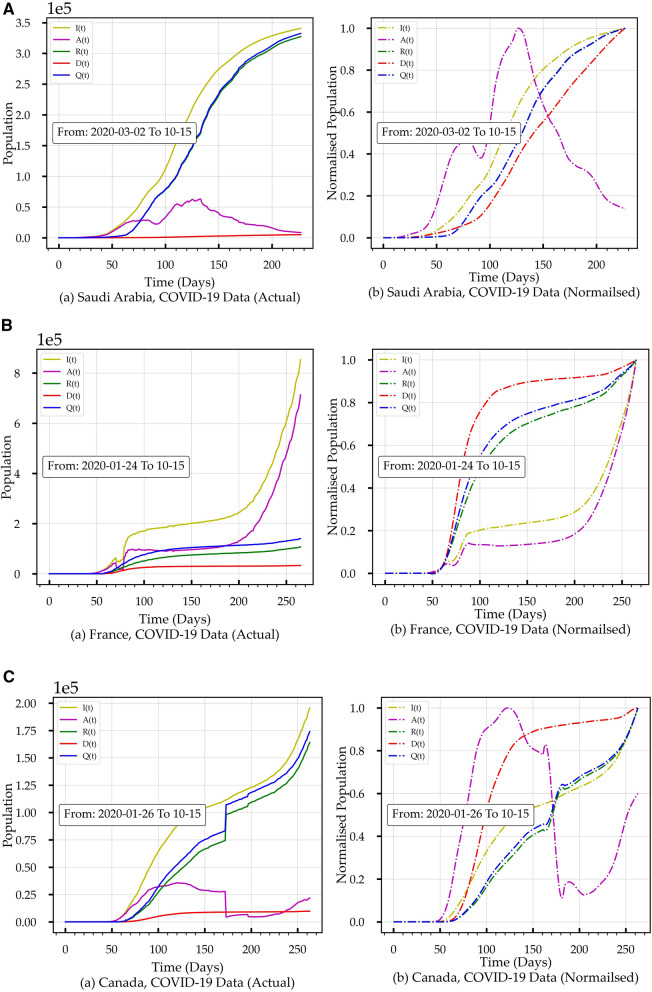
(**A**) Two versions of the COVID-19 published data with the end date of October 15th, 2020 for Saudi Arabia. (**a**) the actual uploaded data. (**b**) the normalised versions of the data. (**B**) Two versions of the COVID-19 published data with the end date of October 15th, 2020, for France. (**a**) the actual uploaded data. (**b**) the normalised version of the data. (**C**) Two versions of the COVID-19 published data with the end date of October 15th, 2020 for Canada. (**a**) the actual uploaded data. (**b**) the normalised version of the data.

The data set for Saudi Arabia, France, and Canada in Fig. 2 includes *I(t)*, Active *A(t)* (hospitalised population rate *H(t)* plus *Q(t)*), *R(t)* and *D(t)* stages. Figure 2Aa,Ba,Ca show the actual uploaded data with the end date of October 15th, 2020, while Fig. 2Ab,Bb,Cb show the normalised and smoothed data version for each country. In Fig. 2, the normalised data indicates that the *A(t)* is in decline for Saudi Arabia, on the rise for France, and on the rise after a decline for Canada. The *I(t)* has just started to plateau in Saudi Arabia while still rising in France and Canada. Although *R(t)* is increasing for all countries, it is noticeable that *R(t)* for Canada has a jump around the 175th day.

**Figure 3:**
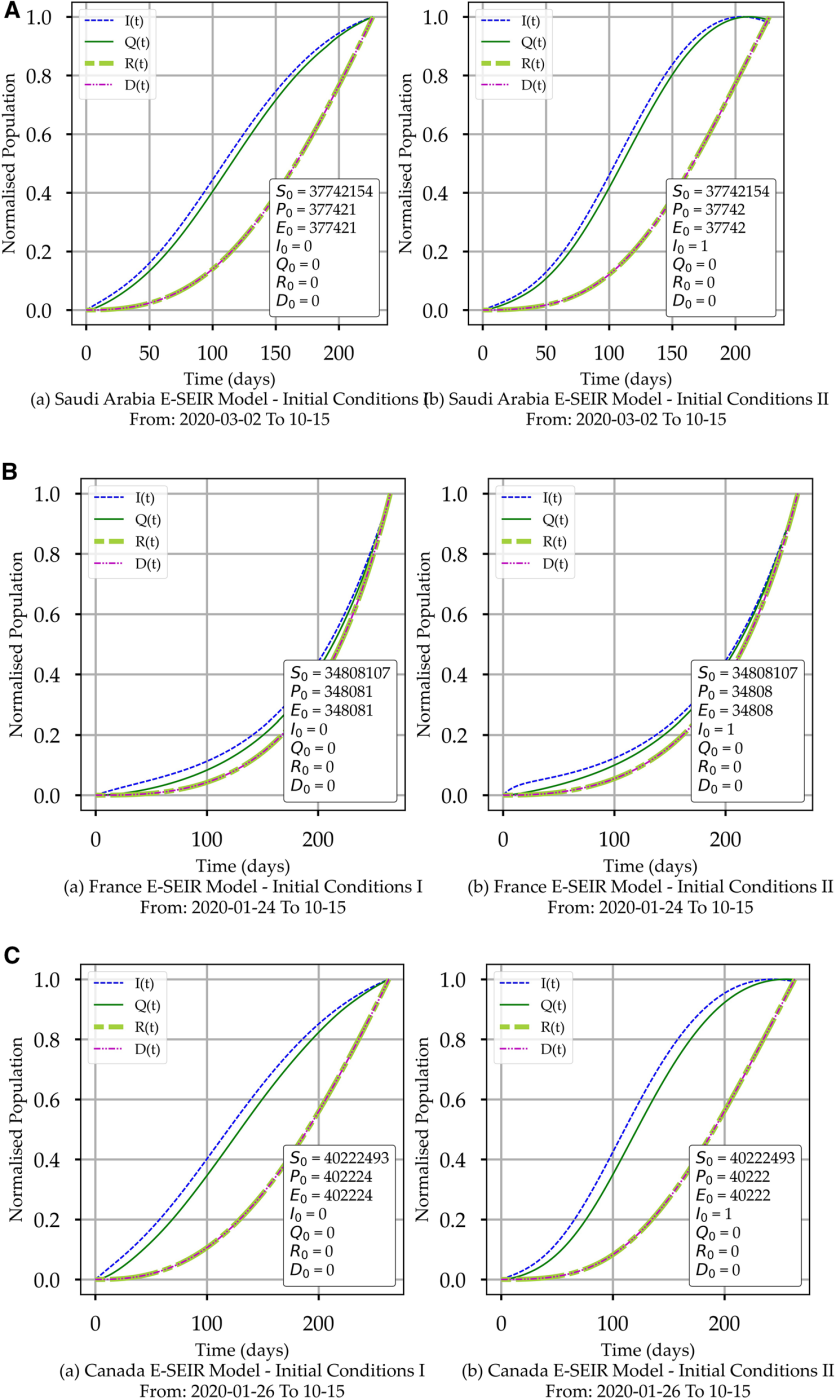
(**A**) COVID-19 E-SEIR Models for Saudi Arabia. (a) initial conditions I. (b) initial conditions II. (**B**) COVID-19 E-SEIR Models for France. (a) initial conditions I. (b) initial conditions II. (**C**) COVID-19 E-SEIR Models for Canada. (a) initial conditions I. (b) initial conditions II.

From Figs. 2 and 3, it is easy to observe within the actual data space and model space, respectively, the changes in *I(t)*, *A(t)* and *R(t),* which reflect the cumulative impact of the health controlling efforts (HCE) exerted by the public and by governments. No research group has managed to quantify and deduce a function that represents the HCE to the best of our knowledge. Later in “Convolutional prediction model (CPM)—new proposal”, we will show how to conclude the associated HCE profiles from *I(t)*, *A(t)* and *R(t).*

### E-SEIR model computation

Most of the traditional optimisation techniques are centred around evaluating the first derivatives to locate the optima on given limitations in a 2D space. Typically, there is no guarantee of locating the first derivatives in our scenario, so finding the optima for many rough and discontinuous optimisation surfaces will not be safe. Hence, we need to use derivative-free optimisation algorithms. Such an approach is referred to as an intelligent search problem, where one or more computational agents are utilised to locate the optima on a real-value search space with its embedded set of initial conditions for the core optimisation problem^22^. The most well-known algorithms are particle swarm optimisation (PSO) which is used by^10^, and differential evolution (DE) that we used in this work. The DE method is a special kind of differential operator which can be invoked and easily implemented with minor parameter tuning^22^. We collected the values of these parameters that govern the SoDE via a callback function of a defined signature that we pass as one of the Python DE library optimisation call attributes. In turn, this callback function populates a global/static n-dimensional array, where n is the number of parameters in the SoDE. The DE library optimisation call also allows us to select one of three optimum search strategies. In our computation, we used 'best1bin' as the optimum search strategy.

To solve Eqs. (6-12) and build the models shown in Fig. 3, we downloaded the actual data of COVID-19 for each country with the end date October 15th, 2020 from^25^ coupled with two sets of initial conditions shown in Table 1. It is worth mentioning that when we used normalised COVID-19 data, the solution model exhibited a peak with a considerable shift to the left, i.e., much earlier than the actual, rendering the solution unreliable; hence it is not included in the paper. In the model discussion, we will focus on the *I(t)* profile. We will cover other E-SEIR rate components when necessary because we are using the *I(t)* as the leading indicator in our comparison with the new CPM model. We did set the following value limits 0 ≤ p ≤ 1, where p is any of the parameters α, β, γ, δ, λ, or κ in Eqs. (6-12). Each run takes around 45–60 min to execute the computation of a model for each country using dual (four logical) processors, 2.7 GHz, and 16 GB memory with Windows 10 Pro operating system.

**Table 1:** Two sets of initial conditions.

Initial stage	Initial conditions I	Initial conditions II
*S*_*0*_	Country population	Country population
*P*_*0*_	1.0% of Country population	0.1% of Country population
*E*_*0*_	1.0% of Country population	0.1% of Country population
*I*_*0*_	0	1
*Q*_*0*_	0	0
*R*_*0*_	0	0
*D*_*0*_	0	0

Initial conditions I imply that the infection is triggered via the higher *E*_*0*_ percentage, hence *I*_*0*_ = 0. Initial conditions II imply that the infection is already triggered. i.e., *I*_*0*_ = 1 with lower *E*_*0*_ percentage.

From Figure 3, we summarised the trend for *I(t), Q(t)*, *R(t), and D(t)* in Table 2. It is easy to observe the differences in the E-SEIR component profile depending on the initial conditions. This is an inherent attribute in the E-SEIR formulation and its computation.

**Table 2:** E-SEIR model curves trends summary for two initial conditions sets for Saudi Arabia, France, and Canada.

Country	E- SEIR stage	Model trend Initial conditions I	Model trend Initial conditions II
Saudi Arabia	*I(t)*	Continuous rise	Peaked and has just started its decline
	*Q(t)*	Continuous rise	Has just begun its plateau
	*R(t)*	Continuous rise	Continuous rise
	*D(t)*	Has just started its decline	Continuous decline
France	*I(t)*	Continuous rise	Continuous rise
	*Q(t)*	Rapid rise	Rapid rise
	*R(t)*	Rapid rise	Rapid rise
	*D(t)*	Rapid rise	Rapid rise
Canada	*I(t)*	Continuous rise	Has a short plateau, just started its decline
	*Q(t)*	Rapid rise	Has just begun its plateau
	*R(t)*	Rapid rise	Continuous rise
	*D(t)*	Rapid rise	Continuous rise

### The role of the E-ESIR model equation parameters

**Figure 4:**
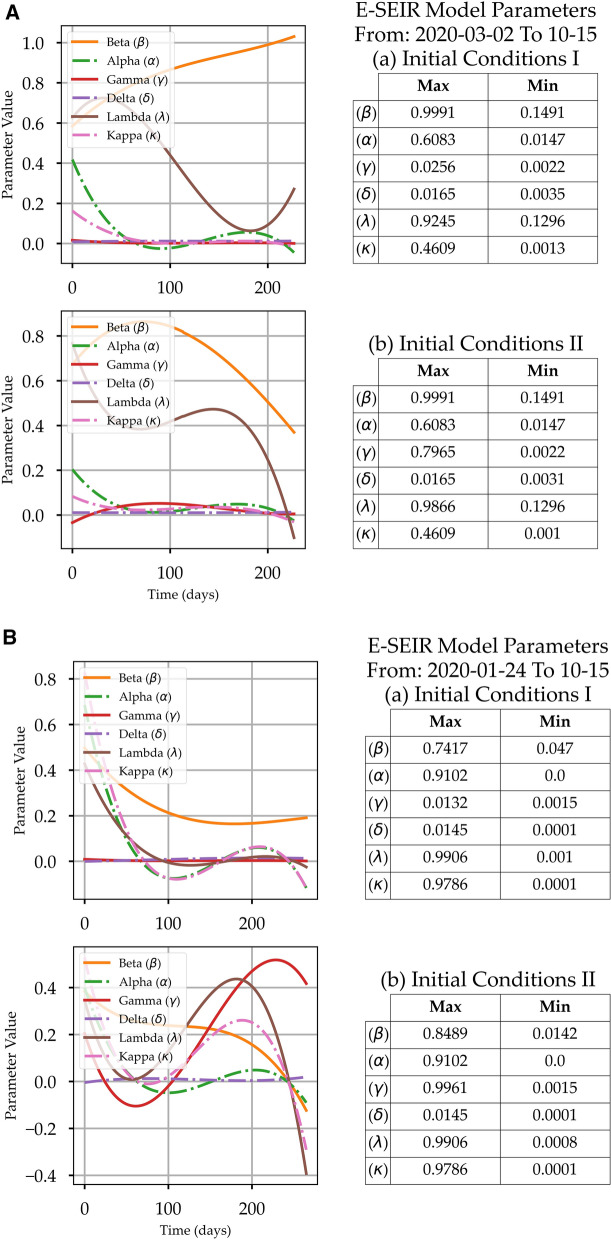

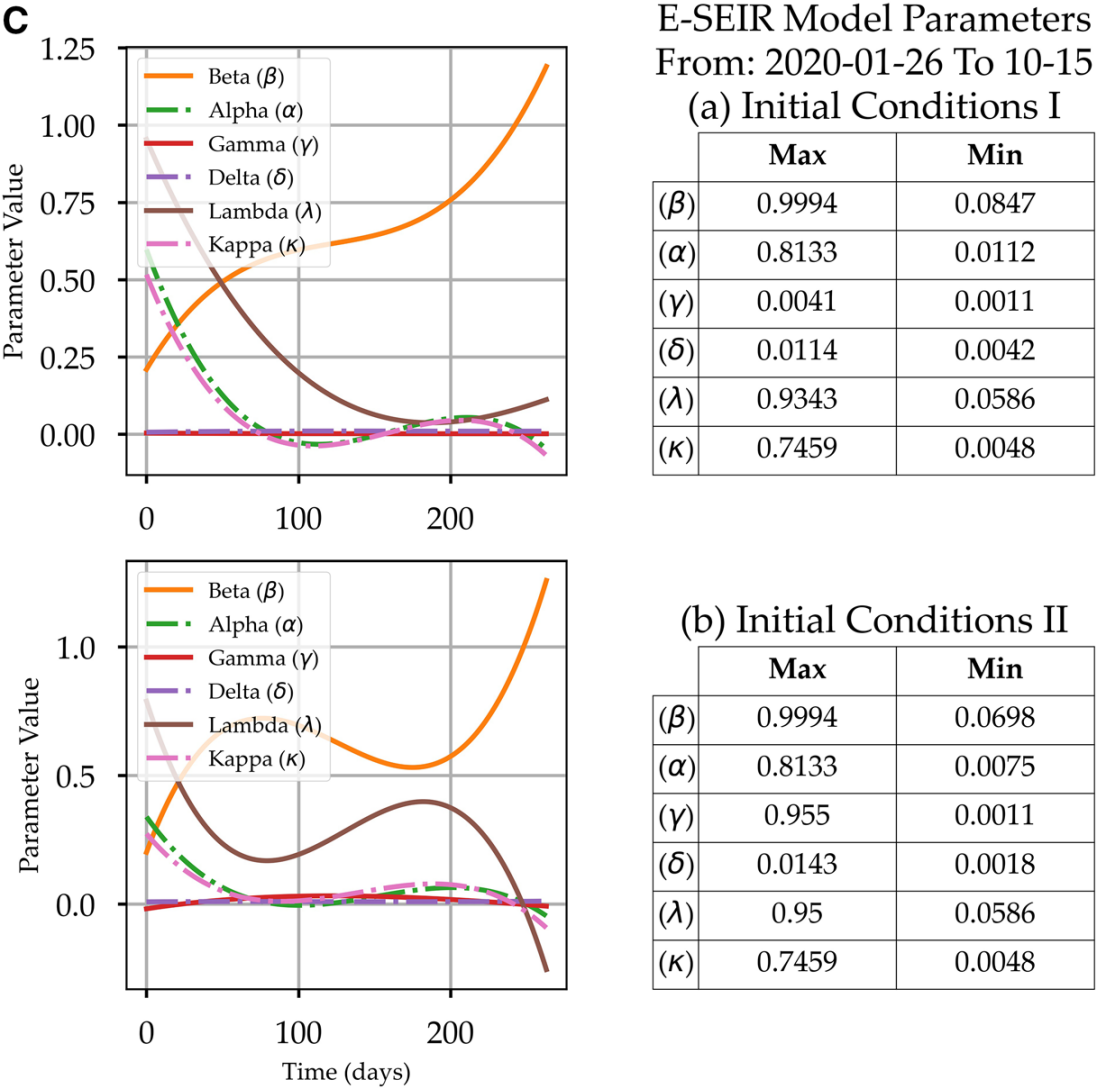
(**A**) The E-SEIR Models' Solution Parameters α, β, γ, δ, λ, and κ Profiles and Maximum and Minimum Value Sets for two sets of initial conditions for Saudi Arabia. (**B**) The E-SEIR Models' Solution Parameters α, β, γ, δ, λ, and κ Profiles and Maximum and Minimum Value Sets for two sets of initial conditions for France. (**C**) The E-SEIR Models' Solution Parameters α, β, γ, δ, λ, and κ Profiles and Maximum and Minimum Value Sets for two sets of initial conditions for Canada.

Before concluding the predictions from these models, it is worth reviewing the behaviour of the optimum solution parameters α, β, γ, δ, λ, and κ, which are defined in “SEIR and E-SEIR models—an overview”. In this study, we have not assumed any function profile for the parameters in Eqs. (6-12) as^12^ did by adopting a specific profile for the *D(t)* rate and the *R(t)* rate. The parameter extraction callback function in the SoDE (“E-SEIR model computation”) allowed us to collect the parameter value sets, from which the maximum and minimum were concluded and shown in Fig. 4. Interestingly, the first column in Figs. 4A–C shows the behaviour profile for each parameter in Eqs. (6-12). The parameters' change behaviour could be a good indicator of the reliability of the numerical solutions. Some of the parameters' curves show unexpected behaviours that could be attributed to the smoothing steps that might mask the actual underlying profile, the initial conditions that might shift the real peaks' locations, and the optimisation technique that requires more consistent input data. Investigating such issues is necessary to increase confidence in the model outcomes. Such investigation is beyond the scope of this work because the scope of this work is to establish a comparison between the E-SEIR model and our proposed CPM model in terms of (1) required resources, (2) efforts, (3) complexity, and (4) scope of the results, i.e., peak predictions and health efforts. The beta (β) profile has the expected decreasing profile for Saudi Arabia, France, and Canada with initial conditions I. For initial conditions II, the β profile shows unexpected fluctuations with an increasing trend, which does not coincide with the model. Delta (δ) profile shows a small decreasing trend for Saudi Arabia and Canada, which means fewer people will quarantine. For France, the δ profile fluctuates between a minimum and maximum and records the highest rate with initial conditions II. It can be useful to overview the values of the main parameters to define a realistic range of values and understand their impact on the model. The κ profile for all countries' models has an exponential decreasing profile as has been assumed by^23^, which means that the death rate is decreasing. The κ profile does not coincide with the *D(t)* rising trend for the actual COVID-19 data shown in Fig. 2, which indicates (1) there is bias in the E-SEIR model formulation, (2) the assumption of initial conditions is not realistic, or (3) the suggested parameter value limits are not correct.

### E-SEIR model predictions

**Figure 5:**
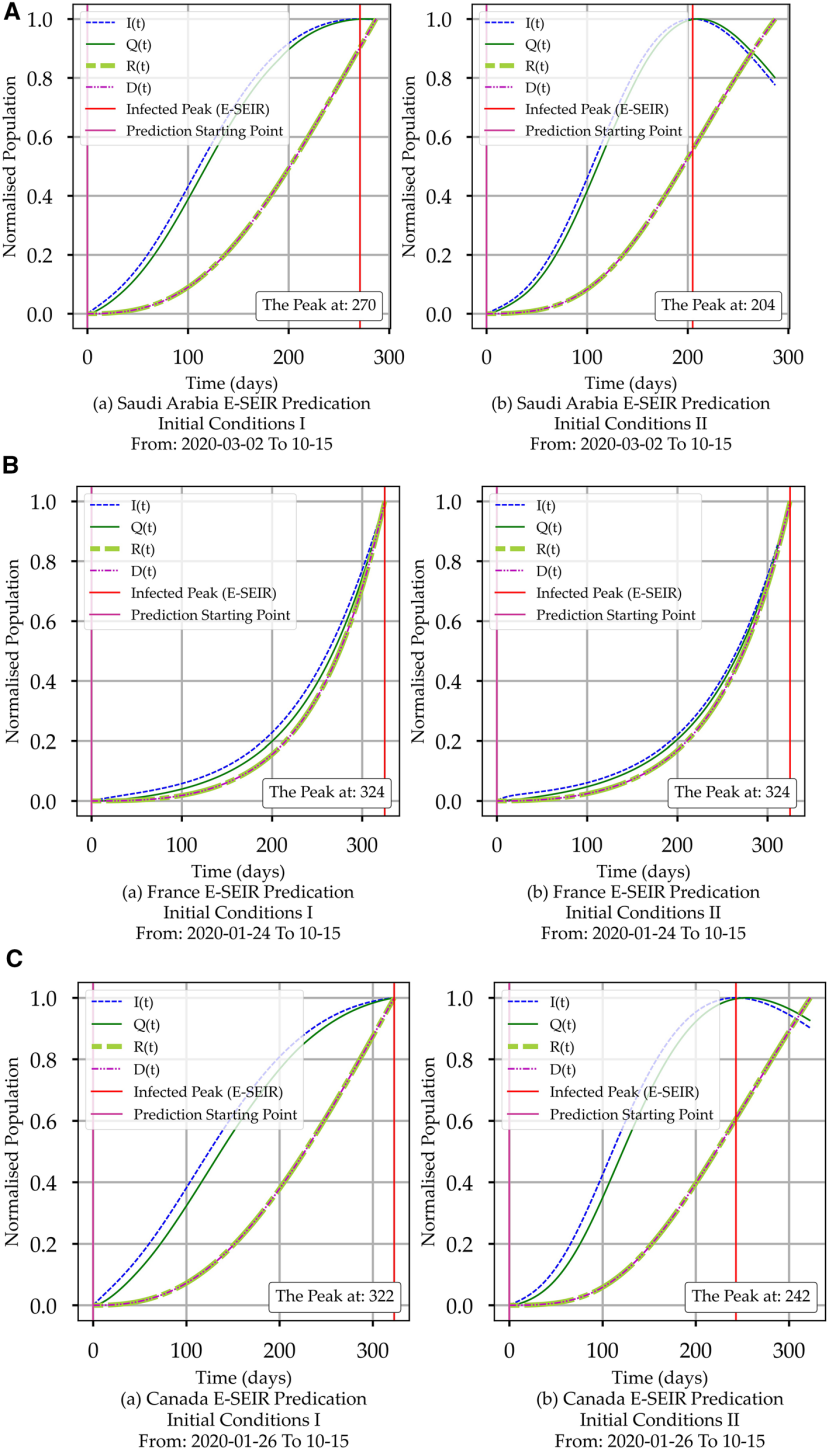
(**A**) The E-SEIR Model Predictions for Saudi Arabia. (a) shows the predictions associated with initial conditions set I. (b) shows the predictions related to initial conditions set II. (**B**) The E-SEIR Model Predictions for France. (a) shows the predictions associated with initial conditions set I. (b) shows the predictions associated with initial conditions set II. (**C**) The E-SEIR Model Predictions for Canada. (a) shows the predictions associated with initial conditions set I. (b), which is related to initial conditions set II.

Figure 5 shows the deterministic prediction profiles for the E-SEIR model of each country. The first column shows the model, and the second the deterministic prediction for 60 days beyond the uploaded COVID-19 data. The model profiles for *I(t), A(t), Q(t), R(t)* and *D(t)* are concluded by using mainly the DE python numerical library with the same initial conditions for all three countries. This assumption is safe to avoid any potential bias in numerical computing of the optimum model solution for Eqs. (6-12). Hence, only the actual COVID-19 data for each country will be the exclusive driver to reach an optimum solution based on which we collected the parameters' values. We have applied two sets of boundary values listed in Table 1, resulting in a minimal model difference. Initial conditions I imply that the infection will be triggered via the higher *E*_*0*_ percentage. Hence *I*_*0*_ can be set to 0. Initial conditions II suggest that the infection is already triggered; hence *I*_*0*_ can be set to 1 with a lower *E*_*0*_ percentage. The difference between the parameters' values is highly noticeable but with the same functional profile trends.

It is interesting to report that using normalised and smoothed COVID-19 data with the DE numerical Python library produces an unexpected shift to the left for the peak value of *I(t).* A similar shift appears for *A(t), Q(t), R(t)* and *D(t)* curves. The summary of E-SEIR stage curve trends the model predictions for each country are shown in Table 2. The prediction time range is 60 days beyond the uploaded data date: the reason we do not exceed 60 days is that we believe that the prediction time range should not be more than 50% of the actual data span in days as it could cause solution divergence which we want to avoid. Time ranges of 60 days are more practical.

## Epidemic process framework components

**Figure 6:**
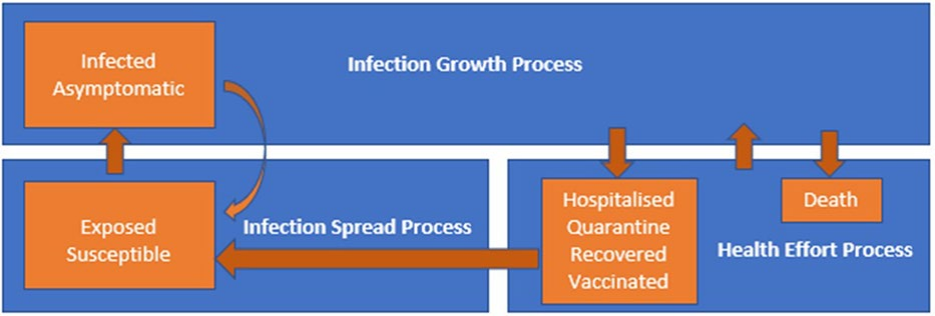
Epidemic Process Framework shows the topology and dependencies of the three main processes: Infection Spread Process (ISP), Infection Growth Process (IGP), and Health Efforts Process (HEP), which also includes process interaction channels.

The epidemic spread and growth models describe the temporal stage-population patterns of disease outbreaks within a geographical area, which help to comprehend the factors that influence infection cases. Modelling is a critical tool in understanding what course of treatments and interventions can be most effective: (1) in terms of time and location, (2) how fast and cost-effective these approaches may be, and (3) what specific factors need to be considered when trying to mitigate and eradicate the disease. Hence, these findings help to define better strategic and health policies for deploying practical and valuable public health control efforts. From the epidemic process framework (Fig. 6) perspective, we consider these modelling tools as (1) solutions that depict the transitions between infection cycle stages within the infection spread process (ISP) and (2) infection growth process (IGP), leaving the health efforts process (HEP) outside the solution scope. Consequently, there is uncertainty about the effectiveness of health controlling efforts on epidemic progression within each region, province/state, or country and how long controls should remain in place. Hence, it is critically important to define the current controlling effort level, the spread dynamics, the growth of COVID-19 outbreaks, and provide near accurate predictions.

The new Convolution Projection Model (CPM) is for the HEP process component, which is part of the proposed epidemic process framework shown in Fig. 6. In this study, we are using the CPM model to draw the predictions for the populations of infected *I(t)* and active cases *A(t)*. The IGP process represented the growth of the populations of asymptomatic *M(t)* and infected *I(t)*, which are the feeders for the populations of the Hospitalisted *H(t)*, Quarantined *Q(t)*, Recovered *R(t)*, and dead *D(t)*. In the ISP process, the populations of the susceptible *S(t)* and exposed *E(t)* act as feeders to *I(t),* which provides a feedback component to *E(t)*. The HEP process manages the treatment components *H(t)* and *Q(t)*, vaccination component *V(t)*, and the terminating components *R(t)* and *D(t)*. Hence, the available epidemic public data are sufficient without hypothetical initial conditions and control parameters.

## Convolutional prediction model (CPM)—new proposal

“SEIR and E-SEIR models—an overview” reviewed the E-SEIR model's foundation based on Eqs. (6-12), including two sets of population rates as independent variables. The first known-value set covers the following: *I(t)*, *R(t)*, *Q(t)*, and dead *D(t)*. These variables are typically confirmed and published^25^ and are among the solution model's accuracy foundations. The second set includes the following: *S(t)*, *E(t)*, and asymptomatic *M(t)*, which typically are estimated based on an established and recognised approach linked to the age distribution, the level of health services provided in each country, and possibly on the DNA groups. The *S(t)*, *E(t)*, and *M(t)* set is a source of uncertainty for the optimum solution^13^. The *M(t)* is subject to many assumptions that have not yet been formalised, leading to a potentially high uncertainty level that will influence the optimal solution's accuracy. *M(t)* will make the solution comprehensive but not safe because no reliable studies quantify the *M(t)* dependencies. With such multi-faceted uncertainties, it would be worthwhile to explore other modelling approaches which are inherently independent of *S(t)*, *E(t)*, and *M(t).*

The studies in^28,29^ and^31^ tackled the direct association of the epidemic spread and the health control measures without stating clearly the epidemic process framework. The team in^28^ suggested a compartmental epidemic model of COVID-19 for infection prediction and control. The short-term projections show that the model captures the decreasing trend of new COVID-19 infections. Also, the same model reflects that good management of quarantined individuals is more effective than isolated individual management in reducing the disease burden. In^29^, the SEIQR difference-equation model of COVID-19 developed by Li, Ming-Tao et al. considers the transmission with discrete-time imported cases for risk assessment and analysis. The research team noticed the influence of Wuhan and Shanxi city lockdown date, as one of the health effort instruments, on the final scale of new cases. Reference^2^ team used the Markov-Chain Monte-Carlo method to determine the model parameters. Sun, Gui-Quan et al. in^31^ developed dynamical models to inspect the COVID-19 spread in Wuhan city to conclude pandemic spread mechanisms. The research explored the impact of lockdown and medical resources as health efforts to control the spread. One of the main model findings is that with the subsequent lockdown imposed by Wuhan authorities, the fewer of the population will be infected regionally and across the country. However, the importance of the findings in^28,29^ and ^31^is related to the fact that research outcomes help (1) the authorities to quantify the impact of their health control steps on the COVID-19 spread and (2) avoiding the harsh impact on the people's movements and subsequent economics. Such a driver is also one of the objectives of our new CPM model, which we are presenting in the next section.

### CPM formulation

**Figure 7:**
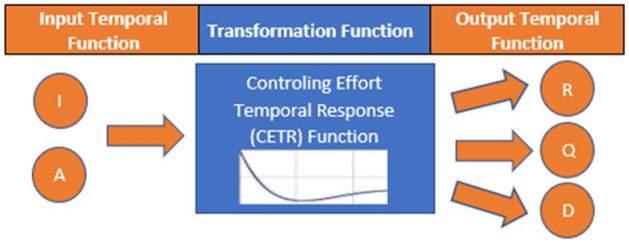
Convolutional prediction model (CPM).

In this section, we are going to present the foundations of the Convolutional Prediction Model (CPM) to determine the projection of *I(t)* and *A(t)*, which is equal to *H(t)* + *Q(t)*, beyond the end date of the collected COVID-19 data. As shown in Fig. 7, the CPM model is based on the transformation caused by the controlling effort temporal response (CETR) that corresponds to the health efforts over a period. The CETR impulse response converts the input temporal functions of *I(t)* and *A(t)* into output temporal functions of *R(t), Q(t)* and *D(t)*. To conclude CPM formulation, the first step is to re-format the relationship between the SEIR stages based on collected data that represent real-valued time-dependent variables which can be described as:

13 $$I\left( t \right)\, = \,i_{1} H\left( t \right)\, + \,i_{2} Q\left( t \right)\, + \,i_{3} R\left( t \right)\, + \,i_{4} D\left( t \right)\, = \,i_{12} A\left( t \right)\, + \,i_{3} R\left( t \right)\, + \,i_{4} D\left( t \right)$$

H(t) and Q(t) represent the number of COVID-19 patients in hospitals and quarantine. *R(t)* and *D(t)* are as defined in the SEIR model. In Eq. (13), {0 < i_n_ ≤ 1} is a set of reporting accuracy factors with a maximum value of 1 which means fully reported data. *A(t)* is the active cases (Active) and is given as (*i*_*1*_* H* + *i*_*2*_* Q*). In Eq. (13), *I(t), H(t), Q(t), R(t)*, *D(t)* and *A(t)* will be referred to as *I, H, Q, R*, *D,* and A respectively for simplicity. Equation (13) can be described from health controlling efforts temporal response CETR (treatments, isolation, social distancing, face mask, social group size, personal hygiene, etc.), as shown in Table 5. This table defines the epidemic progression levels: infection, treatment, and the outcome (Recovery or Death). In this manner, we are avoiding the uncertainty portion of the epidemic cycle, focusing on most of the IGP and HEP processes.

**Figure 8:**
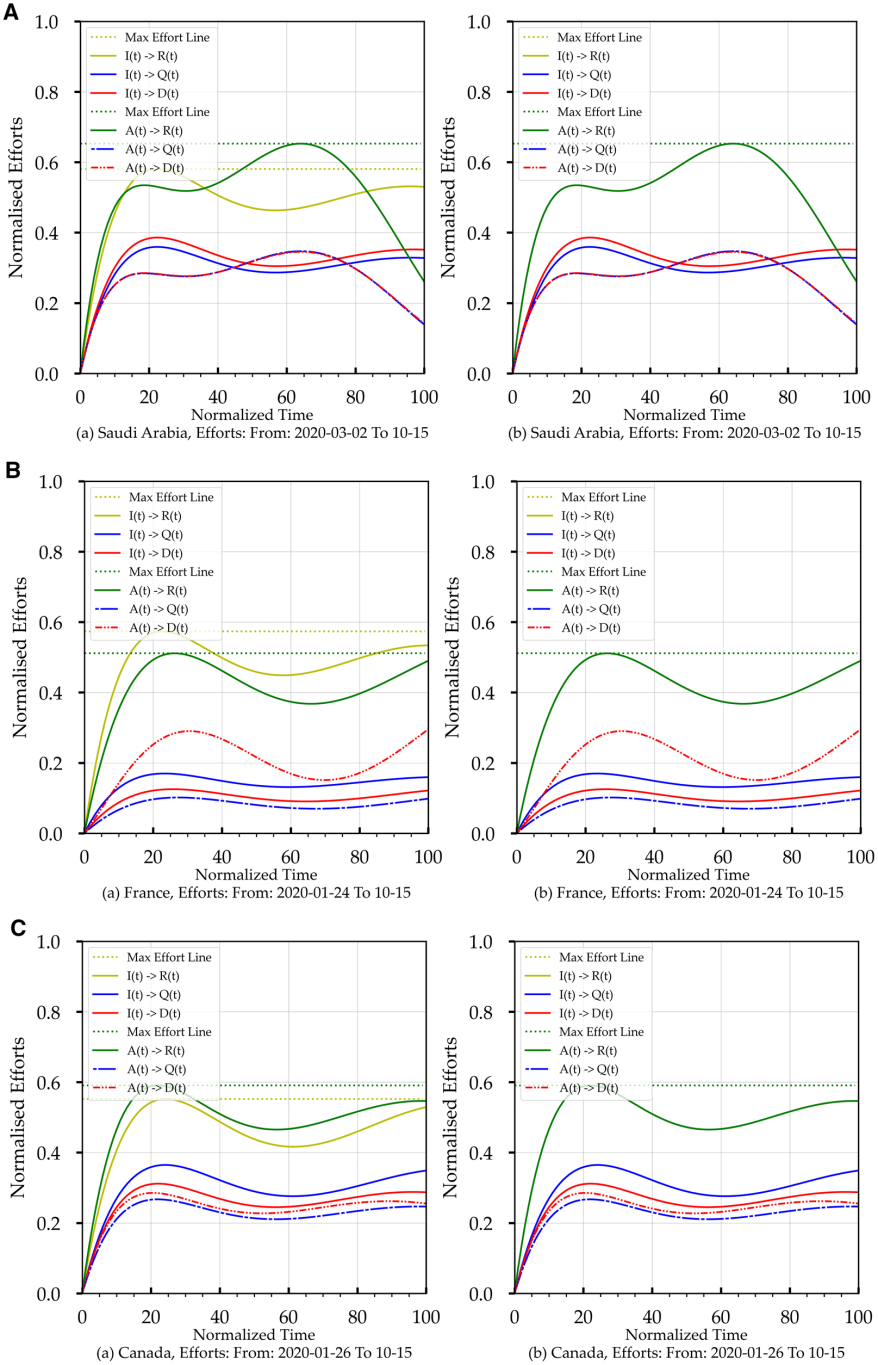
(**A**) The Increasing Version of the Health Efforts Function (CETR) for Saudi Arabia, Source Stages: Infected and Active. (a) covers the period from March 2nd, 2020, to October 15th, 2020. (b) covers the period from March 2nd, 2020 to December 2nd, 2020. (**B**) The Increasing Version of the Health Efforts Function (CETR) for France, Source Stages: Infected and Active. (a) covers the period from March 2nd, 2020, to October 15th, 2020. (b) covers the period from March 2nd, 2020 to December 2nd, 2020. (**C**) The Increasing Version of the Health Efforts Function (CETR) for Canada, Source Stages: Infected and Active. (a) covers the period from March 2nd, 2020, to October 15th, 2020. (b) covers the period from March 2nd. 2020 to December 2nd, 2020.

Table 4 shows that each arrow represents a continuous monotonically increasing or decreasing CETR function, which causes the transition between marked stages on the two sides of the transition arrow. Accordingly, Eq. (13) can be converted from a number relationship function into the corresponding CETR transition function:

14a $$R\left( t \right)\, = \,f_{IR} \left( t \right) \, * \, I \, \left( t \right)$$

14b $$D\left( t \right)\, = \,f_{ID} \left( t \right) \, * \, I\left( t \right)$$

14c $$A\left( t \right)\, = \,f_{IHQ} \left( t \right) \, * \, (i_{1} H\, + \,i_{2} Q)\, = \,f_{IA} \left( t \right) \, * \, I\left( t \right)$$

14d $$R\left( t \right)\, = \,f_{AR} \left( t \right) \, * \, (i_{1} H\, + \,i_{2} Q)\, = \,f_{AR} \left( t \right) \, * \, A\left( t \right)$$

14e $$D\left( t \right)\, = \,f_{AD} \left( t \right) \, * \, (i_{1} H\, + \,i_{2} Q)\, = \,f_{AD} \left( t \right) \, * \, A\left( t \right)$$

where *I(t)* is the confirmed infected population listed in the country's COVID-19 data, in Eq. (14), * denotes a convolution operation, and *f*_(.)_ represents the CETR for the transition between two stages. So, we can place the CETR functions into two categories based on the source stage: *I(t)* based (*f*_*IR*_*(t)* and *f*_*ID*_*(t)*), and *A(t)* based (*f*_*AR*_*(t), and f*_*AD*_*(t)*). Figure 8 shows the functions for two different reporting periods (a) from March 2nd, 2020 to October 15th, 2020 and (b) from March 2nd 2020, to December 2nd 2020, for the three countries in this study. To obtain these CETR transition functions, the first step is to compute the Fourier Transform (FT) *f*_I,_ *f*_R_, and *f*_D_ for *I(t)*, *R(t)*, and *D(t),* respectively, using the normalised data curves in Fig. 2A-b,B-b,C-b. The second step is to compute the inverse of the FT for (*f*_R/_*f*_I_) and (*f*_D/_*f*_I_), i.e., executing a deconvolution operation, which yields *f*_*IR*_*(t)* and *f*_*ID*_*(t)*. By applying the same steps, we compute *f*_*AR*_*(t)* and *f*_*AD*_*(t)*. Each of these CERT functions represents the cumulative health controlling efforts that produce the outcome of stage transition, as shown in Table 4. It is important to remember that CERT functions are typically monotonically decreasing functions. Still, we are showing the curves of [1- *f*_(.)_/max(*f*_(.)_)] in Fig. 8, which presents increasing CETR functions so we can do the analysis in terms of positive percentages instead of using negative percentages. We should note here that *I(t)* is the overall health efforts while *A(t)* is a subset of the health efforts, including the hospitalisation efforts.

### Using CPM for health efforts trends analysis

We start our discussion first by focusing on *f*_*IR*_*(t)* and *f*_*AR*_*(t)*. Let us begin with health efforts in Saudi Arabia, shown in Fig. 8A. We notice that *f*_*AR*_ (i.e., primarily hospitalisation-based efforts) was lagging *f*_*IR*_ (i.e., overall health efforts including public health efforts) initially but later started to catch up and went higher than *f*_*IR*_. In other words, the hospitalisation reached its maximum of 68% (Table 6) in the second half of the timeline in Fig. 8Aa, which is equivalent to the end of the first half of the timeline in Fig. 8Ab. The *f*_*IQ,*_* f*_*ID,*_* f*_*AQ, and*_* f*_*AD*_ efforts in Fig. 8Aa,b reach near stability after the third quarter of both periods. This health effort's high performance is reflected in Figs. 2A, 3A and 4A, in terms of reaching a peak, plateau and starting to decline. In Fig. 8B, for France, it is evident that French efforts of *f*_*AR*_ are always lower than that of *f*_*IR*_ by about 10% (Fig. 8Ba) then narrow to 2% in the second period (Fig. 8Bb). In Fig. 8Ba, we can observe that *f*_*AR*_ reached a maximum of 52% (Table 5) and *f*_*IR*_ recorded a maximum of 58%, but in Fig. 8Bb for the second period, *f*_*AR*_ reached 68% and *f*_*IR*_ recorded 64% (Table 5). The lower performance of *f*_*AR*_ and *f*_*IR*_ in the first period explains why *I(t)* for France in Figs. 2B, 3B, and 4B has a continuous rise, compared to Saudi Arabia and Canada. In Fig. 8Ca, fAR reached 62% for Canada, and *f*_*IR*_ recorded 58% (Table 5), which is a good performance but not as good as Saudi Arabia. In the second period in Fig. 8Cb, *f*_*AR*_ is not in a steady-state and reflects the second peak of *I(t),* but the good news is that *f*_*AR*_ went to 72% and the *f*_*AR*_ went to 60% (Table 5) to ensure the suppression of the second peak with an upward trend.

### Using CPM for *I(t)* and *A(t)* predications

In Fig. 8, the CETR temporal profile changes over time are mirrored on the temporal profiles of *I(t)*, *A(t), R(t), and D(t).* Based on these observations, and because these CETR functions are real-valued functions, we are going to examine the possibility of utilising *f*_*IR*_*(t)* and *f*_*AR*_*(t)* to predict the *I(t)* behaviour over a period beyond the end date of COVID-19 data. To achieve this, we need first to extend the date range of CETR functions *f*_*IR*_*(t)* and *f*_*AR*_*(t)* to cover the extended date. This step is safe because CETR functions are the influencer components in the transactions shown in Table 4 and not the input, implying that we assume the same known health efforts will continue over a more extended period. The second step is to execute the convolution operation *f*_(.)_ * *I(t)* to compute the future *I(t)* where *f*_(.)_ is *f*_*IR*_*(t)* or *f*_*AR*_*(t)* depending on the purpose of the prediction.

In some cases, we might need curve-smoothing for the convolution output, and if so, this will be the third step. The curve-smoothing involves selecting the right smoothing window size (SWS), ranging from the entire length of the data grid to medium size to exceedingly small size that might eliminate the curve-smoothing. For small SWS and no curve-smoothing, we can still get a solution profile with detailed changes like *I(t)* and *A(t)* shown in Fig. 2 and over the extended period of the CETR function. We used the same machine capacity environment mentioned in “E-SEIR model computational results and discussion” for the E-SEIR model computation. The overall time required to produce the CPM results covering the health efforts profiles with *I(t)* and *A(t)* predictions is less than five minutes.

**Figure 9:**
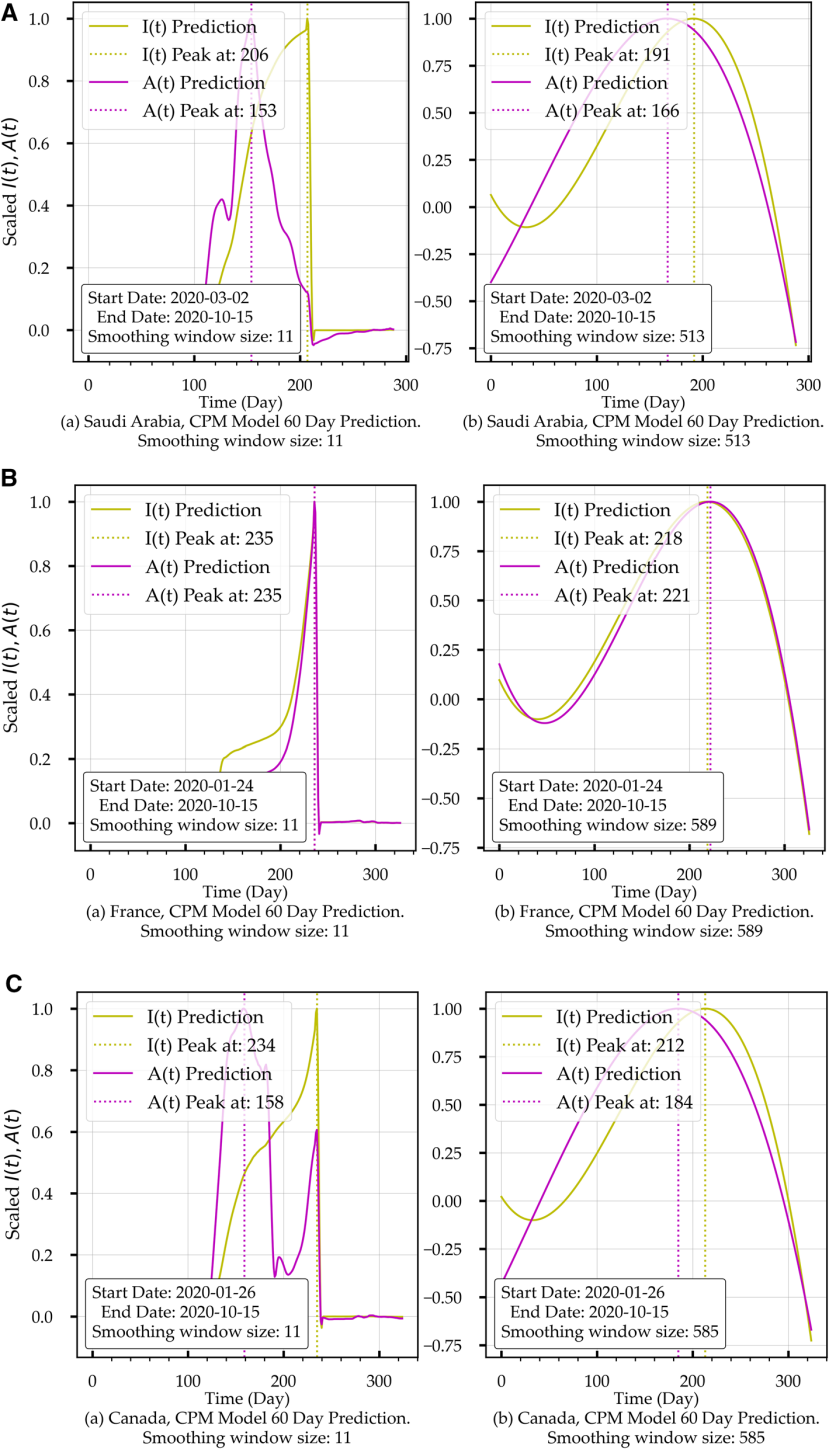
(**A**) Convolution Projection Model (CPM) Predictions for Saudi Arabia. (a) covers the period from 2020-03-02 to 2020-10-15. (b) covers the period from 2020-03-02 to 2020-12-02. (**B**) Convolution Projection Model (CPM) Predictions for France. (a) covers the period from 2020-03-02 to 2020-10-15. (b) covers the period from 2020-03-02 to 2020-12-02. (**C**) Convolution Projection Model (CPM) Predictions for Canada. (a) covers the period from 2020-03-02 to 2020-10-15. (b) covers the period from 2020-03-02 to 2020-12-02.

Figure 9 shows 60 days predictions for *I(t)* and *A(t)* for two smoothing window sizes (SWS). Table 6 shows the E-SEIR and CPM models peak comparison with the corresponding actual COVID-19 data for each country. In Table 6, each red cell indicates that the prediction falls before the corresponding true peak (i.e., not realistic). In contrast, each green cell indicates that the forecast falls after the corresponding actual peak (i.e., realistic). In summary, the CPM predictions for *I(t)* yield 50% realistic predictions when using a small-smoothing window and less than 50% for the large-smoothing window. On the other hand, *I(t)*'s predictions yield 66% realistic predictions with the initial condition I and 100% realistic predictions with initial conditions II.

We can notice that the CPM model (Fig. 9) provides predictions of *I(t)* and *A(t)* while the E-SEIR model (Fig. 5) does not because A(t) is not part of the E-SEIR model's epidemic cycle. From Table 6, we can identify that the initial condition (I) is not the correct assumption for Saudi Arabia, while initial condition II looks suitable for all countries. For the CPM model, *I(t)* predictions for both SWS's fall short compared with the actual data, while A(t) predictions look more promising. Comparing the *I(t)* prediction profiles in Fig. 5 for the E-SEIR model and Fig. 9-(b) for the CPM model with large SWS leads us to conclude that both produce similar general *I(t)* profiles. However, they differ in predicting the time-location of the peaks.

## Discussion and conclusion

Using a 60 + day *I(t)* peak prediction as a primary basis for comparing the E-SEIR and CPM models is insufficient without deploying the models within diverse epidemic environments. Hence, selecting three countries across three continents is essential to challenge both models and explore their strengths and weaknesses.

To establish the accuracy of the E-SEIR model, we need to produce around four sets of artefacts (Figs. 3, 4, 5, and Table 3) to ensure the correctness of the assumptions of the initial conditions, parameter constraints and to verify the reliability of the model predictions. For the CPM model, we need just two sets of artefacts (Figs. 8 and 9) without the need for initial conditions, parameter constraints or complected mathematical modelling. CPM model needs much less computational time than the E-SEIR model using the same moderately powered machine. Consequently, the CPM model can be implemented and deployed in a low-budget health authority office without the need for a big-data specialist.

**Table 3:** E- SEIR model prediction curves trends summary for Saudi Arabia, France, and Canada.

Country	SEIR stage curve	Prediction curve trend Initial conditions I	Prediction curve trend Initial conditions II
**Saudi Arabia**	*I(t)*	Peaked at the 271st day, short plateau, no decline	Peaked at the 204th day, short plateau, then started to decline
	*Q(t)*	Mirroring *I(t)*	Mirroring *I(t)*
	*R(t)*	Continuous rise	Continuous rise
	*D(t)*	Continuous decline	Continuous decline
**France**	*I(t)*	Continuous rise, and a possible peak at the 324th day	Continuous rise, and potential peak at 324th day
	*Q(t)*	Mirroring *I(t)*	Mirroring *I(t)*
	*R(t)*	Continuous rise	Continuous rise
	*D(t)*	Continuous decline	Continuous decline
**Canada**	*I(t)*	Peaked at the 322nd day. and began to plateau	Peaked at the 242nd day, plateaued over 50 days, then started to decline
	*Q(t)*	Mirroring *I(t)*	Mirroring *I(t)*
	*R(t)*	Continuous rise	Continuous rise
	*D(t)*	Continuous decline	Continuous decline

From the E-SEIR model artefacts and Table 6, we can conclude the following: (1) for Saudi Arabia, Fig. 5Aa for initial conditions I, shows that *I(t)* profile has a decline after the peak with incorrect time-location; Fig. 5Ab for the initial conditions II shows that *I(t)* profile has a peak and short plateau, but without declining. (2) for France, Fig. 5Ba,b show no peaks under both initial conditions. (3) For Canada, Fig. 5Ca for the initial conditions (I) shows that *I(t)* profile has a peak and a decline but for initial conditions II. Figure 5Cb shows that *I(t)* has not peaked yet.

From CPM model artefacts (Fig. 8, Tables 4, and 5), we can draw the following conclusions for the CPM model for the three countries: (1) The health efforts (68% for *A(t)* hospitalisation efforts and 58% for *I(t)* containment efforts) of Saudi Arabia ensured better control on the *I(t)* and *A(t)* through a higher level than that of France and Canada (see Table 5). For the period up to December 2nd, 2020, *I(t)* and *A(t)* show a downward trend coinciding with the receding infection rate in Saudi Arabia. (2) French health efforts (Fig. 8B) are still lagging up to October 15th 2020, showing a 52% level for *A(t)* and a 58% level for *I(t).* Yet*,* for the most recent efforts up to December 2nd, 2020, the *A(t)* and *I(t)* both started to rise with a steady trend, so we should anticipate a possible containment of *I(t)*. (3) For Canada, the period before October 15th, 2020 (Fig. 8Ca), *A(t)* was at a level of 58%, but after October 15th, the *I(t)* containment health effort stayed nearly at the same level leading to a second peak in infection rate which explains why *A(t)* went to around 78% with a downward trend.

**Table 4:**
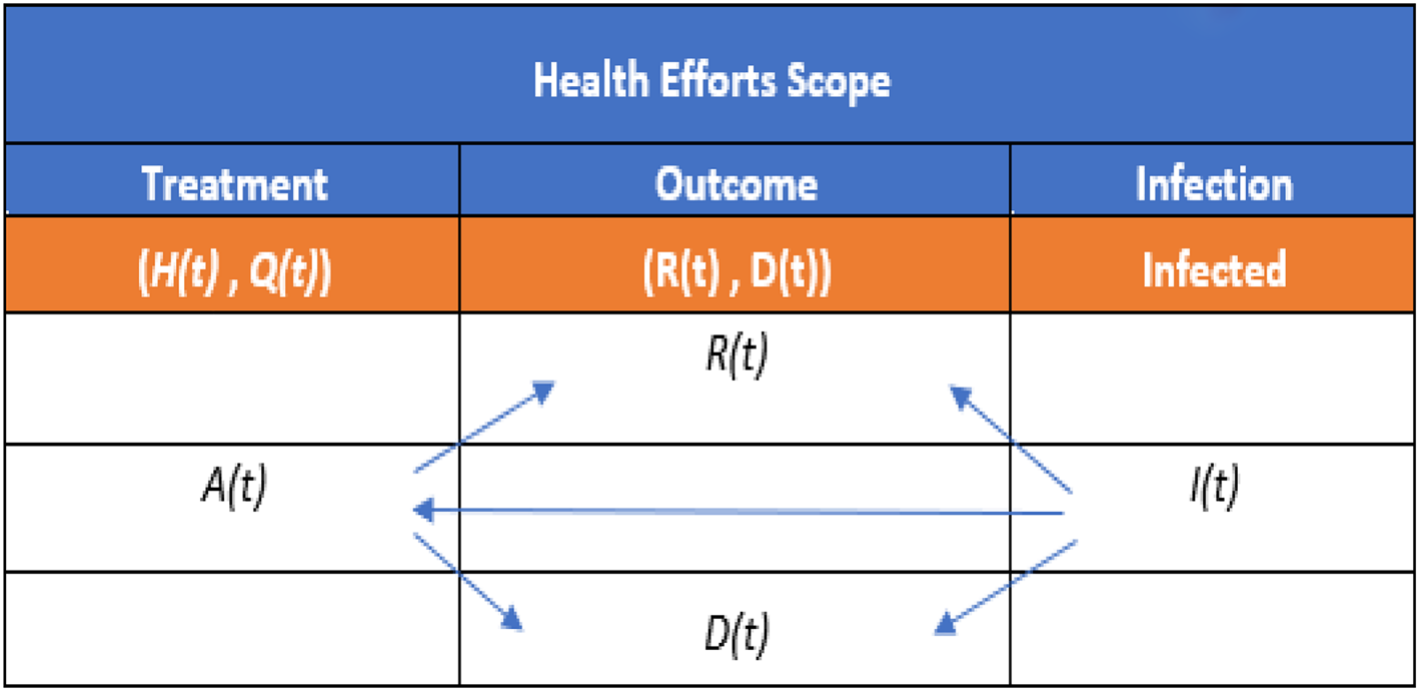
Stage transition links from a health effort perspective.

**Table 5:** CPM model health efforts peaks and moving trends.

Country	Up to OCTOBER 15th 2020			Up to December 2nd 2020		
Health efforts	***I(t)***	***A(t)***	***Trend***	***I(t)***	***A(t)***	***Trend***
Saudi Arabia	58%	68%		55%	66%	
France	58%	52%		66%	68%	
Canada	58%	62%		60%	75%	

Health efforts *I(t)* means the *I(t)* containment efforts. Health efforts *A(t)* implies hospitalisation efforts.

The new CPM model proposed in this paper provides a new tool for health planners to evaluate their controlling efforts' effectiveness. CPM early prediction results indicate that the model provides good A(t) predictions while the E-SEIR model does not offer such predictions. When the right initial conditions are defined, the E-SEIR model provides acceptable *I(t)* predictions that need to be supported with suitable solution parameter profiles.

This paper does not suggest that the CPM model replaces the E-SEIR model but rather complements it. The CPM model can cover the capability gap that the E-SEIR has, assessing the health control efforts as part of the HEP process (Fig. 8 and Table 6) and providing the prediction for *A(t)* related to the hospitalisation capacity within a health region or country.

**Table 6:** E-SEIR and CPM Models peaks predictions time location (in days) comparison.

Country	COVID-19 data (actual)		E-SEIR model		CPM model			
					Small smoothing window		Large smoothing window	
	I(t) Peak	A(t) Peak	I(t) Peak Initial Condition I	I(t) Peak Initial Condition II	I(t) Peak	A(t) Peak	I(t) Peak	A(t) Peak
Saudi Arabia	229	127	**204**	*271*	**208**	*153*	**192**	*164*
France	267	267	*324*	*324*	**237**	*237*	**219**	**222**
Canada	265	125	*265*	*322*	**235**	*159*	**214**	*190*

Each Bold cell indicates that the prediction falls before the corresponding actual peak (i.e., not realistic). In contrast, each italics cell indicates that the prediction falls after the corresponding actual peak (i.e., realistic).

## References

[1] Holmdahl I, Buckee C (2020). Wrong but useful-what Covid-19 epidemiologic models can and cannot tell us. N. Engl. J. Med..

[2] Wang, B. Predictive Model on the Spreading of COVID-19: SEIR. Available online: URL: https://medium.com/@beverly.wang0005/predictive-model-on-the-spreading-of-covid-19-seir-f04f6f2293d5 (accessed on 04 Jul 2020).

[3] Chinazzi M, Davis JT, Ajelli M, Gioannini C, Litvinova M, Merler S, Vespignani A (2020). The effect of travel restrictions on the spread of the 2019 novel coronavirus (COVID-19) outbreak. Science.

[4] Balcan D, Colizza V, Gonçalves B, Hu H, Ramasco JJ, Vespignani A (2009). Multiscale mobility networks and the spatial spreading of infectious diseases. Proc. Natl. Acad. Sci. U.S.A..

[5] Balcan D, Gonçalves B, Hu H, Ramasco JJ, Colizza V, Vespignani A (2010). Modeling the spatial spread of infectious diseases: The GLobal Epidemic and Mobility computational model. J. Comput. Sci..

[6] Fisman D, Khoo E, Tuite A (2014). Early epidemic dynamics of the West African 2014 Ebola outbreak: estimates derived with a simple two-parameter model. PLoS curr..

[7] Zhang Q, Sun K, Chinazzi M, Piontti AP, Dean NE, Rojas DP, Vespignani A (2017). Spread of Zika virus in the Americas. Proc. Natl. Acad. Sci..

[8] Zhu Y, Chen YQ (2020). On a statistical transmission model in analysis of the early phase of COVID-19 outbreak. SIB.

[9] Menni C, Valdes AM, Freidin MB, Sudre CH, ,; Nguyen, L. H., Drew, D. A., Spector, T. D. (2020). Real-time tracking of self-reported symptoms to predict potential COVID-19. Nat. med..

[10] Zhang, Y.; Yu, X.,; Sun, H.,; Tick, G. R.; Wei, W.; Jin, B. COVID-19 infection and recovery in various countries: Modeling the dynamics and evaluating the non-pharmaceutical mitigation scenarios. *arXiv preprint arXiv:* **2020**, *2003.13901*.

[11] COVID, I. H. M. E, (2021). Modeling COVID-19 scenarios for the United States. Nat. med..

[12] Kennedy DM, Zambrano GJ, Wang Y, Neto OP (2020). Modeling the effects of intervention strategies on COVID-19 transmission dynamics. J. Clin. Virol..

[13] Neto OP, Reis JC, ,; Brizzi, A. C. B., Zambrano, G. J., de Souza, J. M., Pedroso, W., Zângaro, R. A. (2020). Compartmentalized mathematical model to predict future number of active cases and deaths of COVID-19. Res. Biomed. Eng..

[14] François L (2020). A brief theory of epidemic kinetics. Biology.

[15] Sana J, James AY (2020). When the best epidemic models are the simplest. Biology.

[16] Annas S, Pratama MI, Rifandi M, Sanusi W, Side S (2020). Stability analysis and numerical simulation of SEIR model for epidemic COVID-19 spread in Indonesia. Chaos Soliton Fract..

[17] Egonmwan AO, Okuonghae D (2018). Analysis of a mathematical model for tuberculosis with diagnosis. J. Appl. Math. Comput..

[18] Abdallah SW, Estomih SM, Oluwole DM (2012). Mathematical modelling of HIV/AIDS dynamics with treatment and vertical transmission. Appl. Math..

[19] Ashley, T.; Jacqueline, S.; John, S. Modeling the spread of tuberculosis in a closed population. Available online: URL: http://educ.jmu.edu/strawbem/math_201/final_reports/Scotti_Takahashi_Spreadbury_Final.pdf **2010**. (accessed on April 20th **2018**).

[20] Godio A, Francesca P, Vergnano A (2020). SEIR modeling of the italian epidemic of SARS-CoV-2 using computational swarm intelligence. Int. J. Environ. Res. Public Health.

[21] Peng L, Yang W, Zhang D, Zhuge C, Hong L (2020). Epidemic analysis of COVID-19 in China by dynamical modelling. MedRxiv Epidemiol..

[22] Das S, Abraham A, Konar A (2008). Particle swarm optimization and differential evolution algorithms: Technical analysis, applications and hybridisation perspectives. Int. J. Comput. Intell. Stud..

[23] Cheynet, E. Generalised SEIR Epidemic Model (Fitting and Computation). Available online: https://it.mathworks.com/matlabcentral/fileexchange/74545-generalized-seir-epidemic-modelfitting-and-computation (accessed on April 29th **2020**).

[24] Li MY, Muldowney JS (1995). Global stability for the SEIR model in epidemiology. Math. Biosci..

[25] GitHub. Available online: URL: https://raw.githubusercontent.com/datasets/covid-19/master/data/countries-aggregated.csv (accessed on October 15th 2020)

[26] Silva PC, Batista PV, Lima HS, Alves MA, Guimarães FG, Silva RC (2020). COVID-ABS: An agent-based model of COVID-19 epidemic to simulate health and economic effects of social distancing interventions. Chaos Soliton Fract.

[27] Parunak, H. V. D.; Savit, R.; Riolo, R. L. Agent-based modeling vs. equation-based modeling: A case study and users' guide. *International Workshop on Multi-Agent Systems and Agent-Based Simulation* (pp. 10–25). Springer, Berlin, Heidelberg. **1998**.

[28] Nadim, Sk Shahid, Indrajit Ghosh, and Joydev Chattopadhyay. "Short-term predictions and prevention strategies for COVID-19: a model-based study." *Applied mathematics and computation* 404 (2021): 126251. 10.1016/j.amc.2021.126251 PMC8015415 33828346

[29] Li M-T, Sun G-Q, Juan Zhang Yu, Zhao XP, Li Li, Wang Y, Zhang W-Y, Zhang Z-K, Jin Z (2020). Analysis of COVID-19 transmission in Shanxi Province with discrete-time imported cases. Math. Biosci. Eng.

[30] Tian H, Liu Y, Li Y, Chieh-Hsi Wu, Chen B, Kraemer MUG, Li B (2020). An investigation of transmission control measures during the first 50 days of the COVID-19 epidemic in China. Science.

[31] Sun G-Q, Wang S-F, Li M-T, Li Li, Zhang J, Zhang W, Jin Z, Feng G-L (2020). Transmission dynamics of COVID-19 in Wuhan, China: effects of lockdown and medical resources. Nonlinear Dyn..

